# The Effect of 10 Most Common Nonurological Primary Cancers on Survival in Men With Secondary Prostate Cancer

**DOI:** 10.3389/fonc.2021.754996

**Published:** 2021-10-06

**Authors:** Mike Wenzel, Luigi Nocera, Christoph Würnschimmel, Claudia Collà Ruvolo, Zhe Tian, Fred Saad, Alberto Briganti, Derya Tilki, Markus Graefen, Andreas Becker, Frederik C. Roos, Felix K. H. Chun, Pierre I. Karakiewicz

**Affiliations:** ^1^ Department of Urology, University Hospital Frankfurt, Frankfurt am Main, Germany; ^2^ Cancer Prognostics and Health Outcomes Unit, Division of Urology, University of Montréal Health Center, Montréal, QC, Canada; ^3^ Department of Urology and Division of Experimental Oncology, Urological Research Institute (URI), Istituto di Ricovero e Cura a Carattere Scientifico (IRCCS) San Raffaele Scientific Institute, Milan, Italy; ^4^ Martini-Klinik Prostate Cancer Center, University Hospital Hamburg-Eppendorf, Hamburg, Germany; ^5^ Department of Neurosciences, University of Naples Federico II Reproductive Sciences and Odontostomatology, Naples, Italy; ^6^ Department of Urology, University Hospital Hamburg-Eppendorf, Hamburg, Germany

**Keywords:** mortality, primary prostate cancer, lung cancer, colon cancer, secondary cancer

## Abstract

**Background:**

This study aims to test the effect of the 10 most common nonurological primary cancers (skin, rectal, colon, lymphoma, leukemia, pancreas, stomach, esophagus, liver, lung) on overall mortality (OM) after secondary prostate cancer (PCa).

**Material and Methods:**

Within the Surveillance, Epidemiology, and End Results (SEER) database, patients with 10 most common primary cancers and concomitant secondary PCa (diagnosed 2004–2016) were identified and were matched in 1:4 fashion (age, year at diagnosis, race/ethnicity, treatment type, TNM stage) with primary PCa controls. OM was compared between secondary and primary PCa patients and was stratified according to primary cancer type, as well as according to time interval between primary cancer *vs.* secondary PCa diagnoses.

**Results:**

We identified 24,848 secondary PCa patients (skin, *n* = 3,871; rectal, *n* = 798; colon, *n* = 3,665; lymphoma, *n* = 2,583; leukemia, *n* = 1,102; pancreatic, *n* = 118; stomach, *n* = 361; esophagus, *n* = 219; liver, *n* = 160; lung, *n* = 1,328) *vs.* 531,732 primary PCa patients. Secondary PCa characteristics were less favorable than those of primary PCa patients (PSA and grade), and smaller proportions of secondary PCa patients received active treatment. After 1:4 matching, all secondary PCa exhibited worse OM than primary PCa patients. Finally, subgroup analyses showed that the survival disadvantage of secondary PCa patients decreased with longer time interval since primary cancer diagnosis and subsequent secondary PCa.

**Conclusion:**

Patients with secondary PCa are diagnosed with less favorable PSA and grade. Even after matching for PCa characteristics, secondary PCa patients still exhibit worse survival. However, the survival disadvantage is attenuated, when secondary PCa diagnosis is made after longer time interval, since primary cancer diagnosis.

## Introduction

The most recent US cancer statistics (2018) indicate over 17 million new cancer diagnoses annually. Of these, almost 9 million were made in men (1–3). In men, prostate cancer (PCa) ranks as first or second most frequently diagnosed cancer. Virtually, all contemporary epidemiological studies addressing PCa survival exclusively focused on primary PCa and excluded patients with prior cancers (4–9). It is particularly of note that an increased risk exists for secondary cancers and especially secondary PCa after prior primary cancers (10–16). However, only three epidemiological SEER-based studies (*n* = 18,225; *n* = 5,987; *n* = 1,457) and one European institutional study (*n* = 1,552) addressed mortality in patients with secondary PCa, after initial diagnosis of another malignancy (17–20). All three studies showed worse survival in secondary PCa patients, relative to primary PCa patients. However, none stratified their analyses according to the most common cancer types. However, primary skin cancer may have a different effect than lung cancer. Moreover, it may also be postulated that the time interval between primary cancer and secondary PCa diagnosis may also affect survival in secondary PCa patients but has not been examined to date.

We addressed these two important unaddressed points within the Surveillance, Epidemiology, and End Results (SEER) registry database and hypothesized that they may impact important survival differences.

## Material and Methods

### Study Population

**Figure 1:**
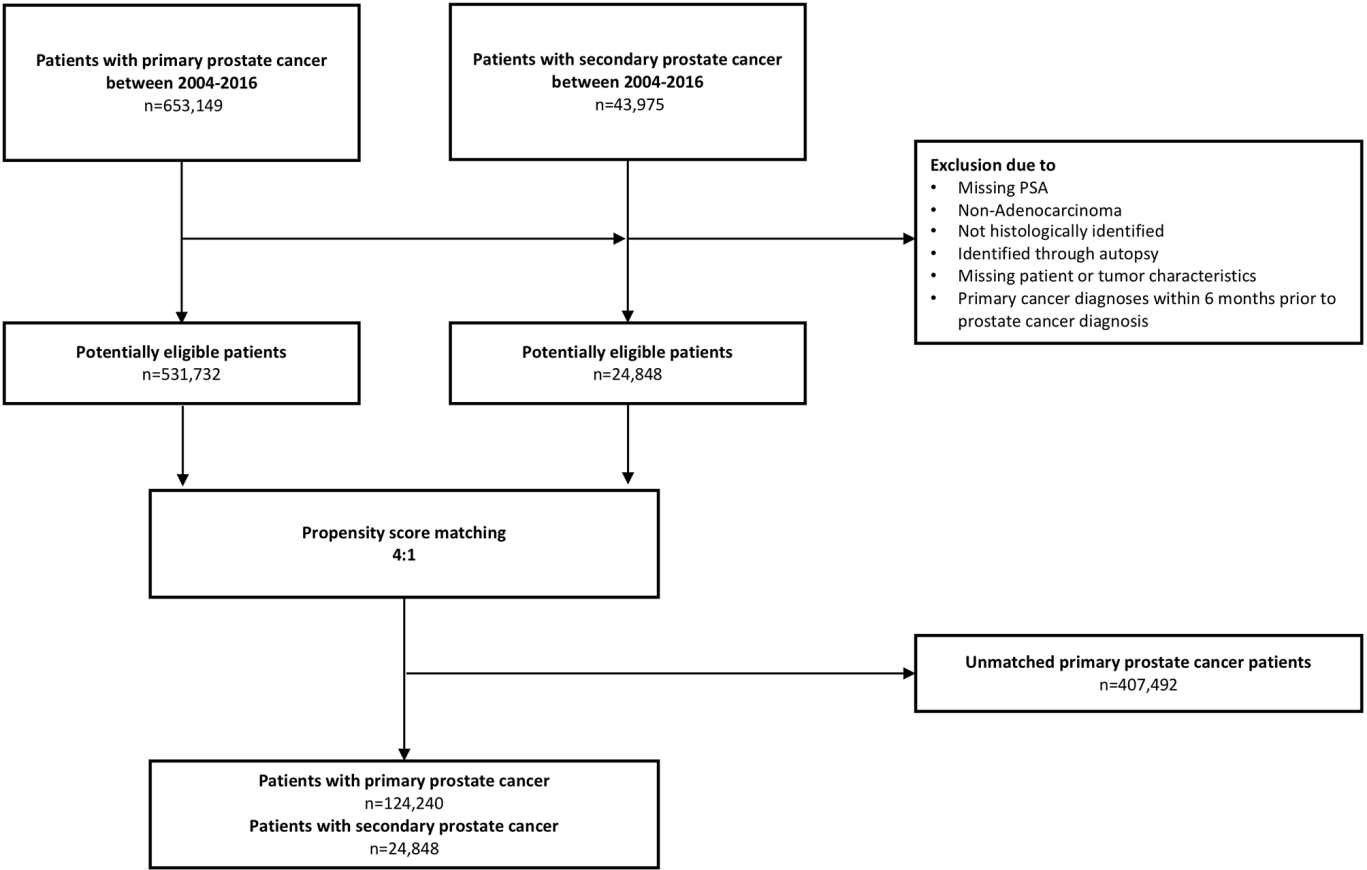
Flow chart depicting included patients with primary and secondary prostate cancer in analyses.

Within the SEER database, we identified all patients ≥18 years old with secondary PCa diagnosed between 2004 and 2016, after prior diagnosis of one of 10 commonest nonurological malignancies (skin, rectal, colon, lymphoma, leukemia, pancreas, stomach, esophagus, liver, and lung). Moreover, we also identified all ≥18-year-old patients with biopsy-proven primary adenocarcinoma of the prostate diagnosed between 2004 and 2016 (International Classification of Disease for Oncology (ICD-O-3) code 8140, site code C61.9). Cases that were identified at autopsy or death certificate or with unknown histology were excluded. Patients with unavailable PSA values were excluded in both cohorts. We excluded concomitantly diagnosed primary cancer and secondary PCa (≤6 months apart), according to previously reported methodology (21, 22). Descriptive statistics addressed all included 24,848 secondary PCa patients and all 531,732 primary PCa patients (**Figure 1**; **Table 1**). Subsequently, survival analyses focused on overall mortality (OM). Here, we relied on a propensity score matched (age at diagnosis, year of diagnosis, race/ethnicity, PCa treatment, cT-stage, cN-stage, and M-stage) cohort of all 24,848 secondary PCa patients that were matched with four primary PCa controls (*n* = 99,392).

**Table 1:** Descriptive characteristics prior to matching and after matching for age at prostate cancer diagnosis, year of prostate cancer diagnoses, race/ethnicity, treatment type, and TNM stage for primary and secondary prostate cancer patients.

Variable		Prior to matching		After matching		
		Primary PCa	Secondary PCa	Overall	Primary PCa	Secondary PCa
		*N* = 531,732	*N* = 24,848	*N* = 124,240	*N* = 99,392 (80%)	*N* = 24,848 (20%)
Age at PCa diagnosis	Median (IQR)	65 (59–72)	69 (64–76)	69 (64–76)	69 (64–76)	69 (64–76)
Year of PCa diagnosis	Median (IQR)	2010 (2007–2013)	2013 (2007–2013)	2013 (2007–2013)	2013 (2007–2013)	2013 (2007–2013)
Age of primary cancer diagnosis	Median (IQR)	–	63 (56–69)	–	–	63 (56–69)
Year of primary cancer diagnosis	Median (IQR)	–	2004 (2000–2008)	–	–	2004 (2000–2008)
PSA (ng/ml)	Median (IQR)	6.6 (4.8–10.6)	6.9 (4.9–11.5)	6.9 (4.9–11.4)	6.9 (4.9–11.3)	6.9 (4.9–11.5)
Follow-up (months)	Median (IQR)	68 (32–104)	53 (23–88)	58 (25–93)	59 (26–94)	53 (23–88)
cT	cT1	324,967 (61.1)	14,719 (59.2)	74,330 (59.8)	59,611 (60.0)	14,719 (59.2)
	cT2	164,054 (30.9)	7,919 (31.9)	40,322 (32.5)	32,403 (32.6)	7,919 (31.9)
	cT3	14,084 (2.6)	635 (2.6)	2,853 (2.3)	2,218 (2.2)	635 (2.6)
	cT4	4,701 (0.9)	248 (1.0)	926 (0.7)	678 (0.7)	248 (1.0)
	cTx	23,926 (4.5)	1,327 (5.3)	5,809 (4.7)	4,482 (4.5)	1,327 (5.3)
cN stage	cN0	493,330 (92.8)	23,026 (92.7)	116,645 (93.9)	93,619 (94.2)	23,026 (92.7)
	cN1	15,055 (2.8)	573 (2.3)	2,295 (1.8)	1,722 (1.7)	573 (2.3)
	cNx	23,347 (4.4)	1,249 (5.0)	5,300 (4.3)	4,051 (4.1)	1,249 (5)
M stage	M0	495,768 (93.2)	23,021 (92.6)	116,431 (93.7)	93,410 (94.0)	23,021 (92.6)
	M1	22,396 (4.2)	1,131 (4.6)	4,834 (3.9)	3,703 (3.7)	1,131 (4.6)
	Mx	13,568 (2.6)	696 (2.8)	2,975 (2.4)	2,279 (2.3)	696 (2.8)
Gleason grade group at diagnosis	I	209,565 (39.4)	8,951 (36.0)	46,422 (37.4)	37,471 (37.7)	8,951 (36.0)
	II	137,937 (25.9)	6,117 (24.6)	30,986 (24.9)	24,869 (25.0)	6,117 (24.6)
	III	60,193 (11.3)	2,968 (11.9)	14,813 (11.9)	11,845 (11.9)	2,968 (11.9)
	IV	46,788 (8.8)	2,548 (10.3)	12,368 (10.0)	9,820 (9.9)	2,548 (10.3)
	V	40,687 (7.7)	2,299 (9.3)	10,795 (8.7)	8,496 (8.5)	2,299 (9.3)
	Unknown	36,562 (6.9)	1,965 (7.9)	8,856 (7.1)	6,891 (6.9)	1,965 (7.9)
D**’**Amico risk group	low	135,502 (25.5)	5,538 (22.3)	29,178 (23.5)	23,640 (23.8)	5,538 (22.3)
	intermediate	210,982 (39.7)	9,892 (39.8)	49,444 (39.8)	39,552 (39.8)	9,892 (39.8)
	high	144,985 (27.3)	7,319 (29.5)	36,118 (29.1)	28,799 (29.0)	7,319 (29.5)
	Unknown	40,263 (7.6)	2,099 (8.4)	9,500 (7.6)	7,401 (7.4)	2,099 (8.4)
Treatment	RP	178,084 (33.5)	5,909 (23.8)	29,099 (23.4)	23,190 (23.3)	5,909 (23.8)
	EBRT	120,891 (22.7)	6,377 (25.7)	32,032 (25.8)	25,655 (25.8)	6,377 (25.7)
	BT	39,655 (7.5)	1,718 (6.9)	9,023 (7.3)	7,305 (7.3)	1,718 (6.9)
	BT+EBRT	21,696 (4.1)	952 (3.8)	4,755 (3.8)	3,803 (3.8)	952 (3.8)
	RP+EBRT	15,121 (2.8)	554 (2.2)	2,684 (2.2)	2,130 (2.1)	554 (2.2)
	RT+RP	156 (0)	8 (0)	33 (0)	25 (0)	8 (0)
	NLT	140,081 (26.3)	8,430 (33.9)	42,278 (34.0)	33,848 (34.1)	8,430 (33.9)
	Unknown	16,048 (3.0)	900 (3.6)	4,336 (3.5)	3,436 (3.5)	900 (3.6)
Chemotherapy	No/Unknown	527,509 (99.2)	24,663 (99.3)	123,432 (99.3)	98,769 (99.4)	24,663 (99.3)
	Yes	4,223 (0.8)	185 (0.7)	808 (0.7)	623 (0.6)	185 (0.7)
Race/ethnicity	Caucasian	363,223 (68.3)	19,536 (78.6)	97,760 (78.7)	78,224 (78.7)	19,536 (78.6)
	African American	81,905 (15.4)	2,758 (11.1)	13,890 (11.2)	11,132 (11.2)	2,758 (11.1)
	Hispanic	48,835 (9.2)	1,494 (6.0)	7,468 (6.0)	5,974 (6.0)	1,494 (6.0)
	Native	1,861 (0.3)	80 (0.3)	340 (0.3)	260 (0.3)	80 (0.3)
	Asian	26,007 (4.9)	948 (3.8)	4,613 (3.7)	3,665 (3.7)	948 (3.8)
	Unknown	9,901 (1.9)	32 (0.1)	169 (0.1)	137 (0.1)	32 (0.1)
Marital status	Married	354,363 (66.6)	17,024 (68.5)	82,781 (66.6)	65,757 (66.2)	17,024 (68.5)
	Unmarried	116,788 (22.0)	5,049 (20.3)	26,519 (21.3)	21,470 (21.6)	5,049 (20.3)
	Unknown	60,581 (11.4)	2,775 (11.2)	14,940 (12)	12,165 (12.2)	2,775 (11.2)
Socioeconomic status	1st quartile	133,678 (25.1)	6,170 (24.8)	32,867 (26.5)	26,697 (26.9)	6,170 (24.8)
	2nd–4th quartile	397,946 (74.8)	18,678 (75.2)	91,373 (73.5)	72,695 (73.1)	18,678 (75.2)
Region	West	270,363 (50.8)	12,440 (50.1)	62,122 (50)	49,682 (50)	12,440 (50.1)
	Midwest	51,705 (9.7)	3,417 (13.8)	13,753 (11.1)	10,336 (10.4)	3,417 (13.8)
	North-East	89,653 (16.9)	4,363 (17.6)	21,531 (17.3)	17,168 (17.3)	4,363 (17.6)
	South	120,011 (22.6)	4,628 (18.6)	26,834 (21.6)	22,206 (22.3)	4,628 (18.6)

### Statistical Analysis

Descriptive statistics included frequencies and proportions for categorical variables. Medians and interquartile ranges (IQR) were reported for continuously coded variables. The Chi-square tested the statistical significance in proportion differences. The *t*-test and Kruskal-Wallis test examined the statistical significance of mean and distribution differences.

The first part of the analyses compared patient and PCa characteristics between all identified secondary (*n* = 24,848) and primary PCa patients (*n* = 531,732). In the second part of the analyses, we focused on overall mortality (OM), after 1:4 propensity score matching. Kaplan-Meier illustrated OM in the overall comparisons, as well as in all subsequent subgroup analyses. Additionally, multivariable Cox regression quantified hazard ratios (HR) that compared secondary *vs.* primary PCa patients, after further adjusting for covariates of the 1:4 matched cohort: PSA, socioeconomic status, Gleason grade group, and D’Amico risk group (all not previously matched). All tests were two sided with a level of significance set at *p* < 0.05 and R software environment for statistical computing and graphics (version 3.4.3) was used for all analyses (23).

## Results

### Descriptive Characteristics of the Study Population Prior to Matching

Prior to matching, 24,848 secondary PCa and 531,732 primary PCa were available for analyses (**Table 1**). Patients with secondary PCa more frequently harbored Gleason grade group IV (10.3% *vs.* 8.8%) and V (9.3% *vs.* 7.7%, *p* < 0.001). Median PSA at diagnosis showed marginal differences between secondary and primary PCa patients (6.9 [IQR 4.9–11.5] *vs.* 6.6 ng/ml [IQR 4.8–10.6], *p* <0.001). In secondary PCa patients, median PSA values at diagnosis of secondary PCa ranged from 6.5 (skin cancer) to 7.8 ng/ml (pancreatic and liver cancer). However, median age at secondary PCa diagnosis was more advanced than in primary PCa (69 *vs.* 65 years, *p* < 0.001). In secondary PCa patients (**Table 2**), median age at secondary PCa diagnoses ranged from respectively 66 (liver cancer) to 72 years (colon cancer). The average time interval between primary cancer diagnosis and secondary PCa diagnosis ranged from 5 (pancreatic, esophagus, liver cancer) to 8 years (skin and rectum cancer). No clinically meaningful differences were recorded in cT-stage, cN-stage, and M-stages between secondary and primary PCa patients. Important differences existed according to use of local therapy [external beam radiation therapy (EBRT) and radical prostatectomy (RP)]. Specifically, in secondary PCa patients, the rate of EBRT was higher (25.7% *vs*. 22.7%) and the rate of RP was lower (23.8% *vs*. 33.5%), relative to primary PCa patients (all *p* < 0.001). In secondary PCa patients, rates of RP ranged from 11.3% (liver cancer) to 29.7% (skin cancer) and rates of EBRT ranged from 19.5% (rectal cancer) to 34.4% (liver cancer).

**Table 2:** Baseline and prostate cancer characteristics of the 10 most common nonurological cancers prior to secondary prostate cancer.

	Median age at primary cancer diagnosis (IQR)	Median age at secondary prostate cancer diagnosis (IQR)	Median PSA at diagnosis in ng/ml (IQR)	RP vs. EBRT treatment (%)	Overall deaths	Died from secondary prostate cancer (%)	Died from primary cancer (%)
**Skin cancer (*n* = 3,871)**	61 (54–69)	69 (63–75)	6.5 (4.8–10.2)	29.7 *vs.* 22.6	749	164 (21.9)	123 (16.4)
**Rectal cancer (*n* = 798)**	62 (55–68)	70 (64–76)	7.6 (5.2–12.7)	20.4 *vs.* 19.5	214	55 (25.7)	40 (18.7)
**Colon cancer (*n* = 3,665)**	65 (58–71)	72 (66–78)	7.7 (5.2–14.0)	17.3 *vs.* 29.1	1,146	215 (18.8)	213 (18.6)
**Lymphoma (*n* = 2,583)**	62 (55–69)	69 (63–75)	6.9 (4.9–11.4)	22.5 *vs.* 27.5	766	123 (16.1)	274 (35.8)
**Leukemia (*n* = 1,102)**	63 (56–70)	69 (64–75)	6.8 (4.9–11.1)	22.2 *vs.* 23.6	340	45 (13.2)	135 (31.4)
**Pancreatic cancer (*n* = 118)**	65 (60–70)	70 (65–74)	7.8 (5.1–13.5)	14.4 *vs.* 26.3	34	6 (17.6)	11 (32.4)
**Stomach cancer (*n* = 361)**	64 (58–71)	71 (65–77)	7.1 (5.0–12.9)	20.2 *vs.* 28.0	118	29 (24.6)	26 (22.0)
**Esophagus cancer (*n* = 219)**	65 (59–69)	70 (65–75)	7.4 (5.1–11.2)	18.7 *vs.* 29.7	74	11 (14.9)	26 (35.1)
**Liver cancer (*n* = 160)**	61 (56–67)	66 (61–71)	7.8 (5.8–12.8)	11.3 *vs.* 34,4	52	11 (21.2)	26 (50.0)
**Lung cancer (*N* = 1,328)**	65 (59–71)	71 (66–76)	7.6 (5.0–12.8)	14.0 *vs.* 31.4	599	59 (9.8)	255 (42.6)
**Overall (*n*=24,848)**	63 (56–69)	69 (64–76)	6.9 (4.9–11.5)	23.8 *vs.* 25.7	4,069	715 (17.6)	1,122 (27.6)

PSA, prostate-specific antigen; RP, radical prostatectomy; EBRT, external beam radiation therapy.

### Survival Analyses After 1:4 Propensity Score Matching

**Figure 2:**
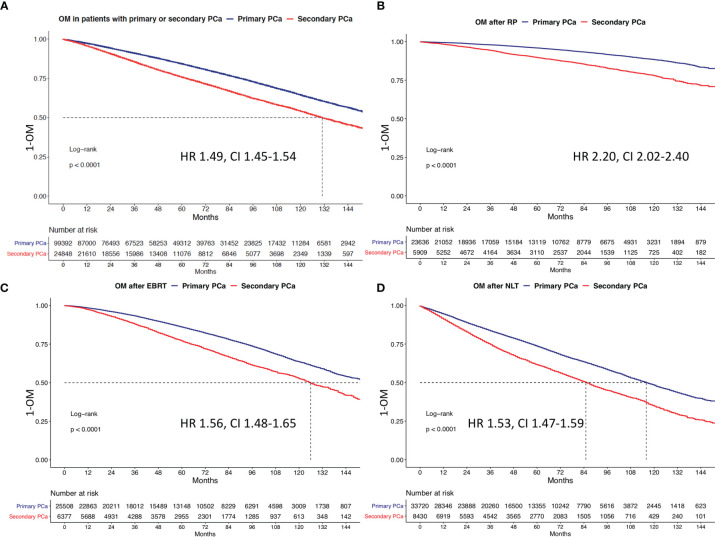
Kaplan-Meier plots depicting overall mortality (OM) for primary and secondary prostate cancer for **(A)** the overall cohort, **(B)** patients treated with radical prostatectomy (RP), **(C)** patients treated with external beam radiation therapy (EBRT), and **(D)** no local treatment (NLT). HR, hazard ratio; CI, confidence interval.

After matching, OM at 10 years was 46.0% in secondary PCa *vs*. 35.7% in primary PCa (**Figure 2A**). The median survival of all 24,848 secondary PCa patients was 131 months and not reached for 99,392 primary PCa patients. This survival disadvantage translated into a 1.49-fold higher risk of OM in secondary PCa patients, relative to their primary PCa counterparts. After further multivariable adjustment, a 1.51-fold higher OM was observed (**Table 3**).

**Table 3:** Univariable und multivariable Cox regression models after adjustment for PSA, socioeconomic status, Gleason grade group, and D’Amico risk stratification.

	Univariable		Multivariable	
	HR (CI)	*p*-value	HR (CI)	*p*-value
**Cancers**				
**Primary prostate cancer**	Ref	–	–	–
**All secondary prostate cancer**	1.49 (1.45–1.54)	<0.01	1.51 (1.47–1.55)	<0.01
**Skin cancer**	1.10 (1.02–1.20)	0.02	1.16 (1.07–1.26)	<0.001
**Colon cancer**	1.22 (1.15–1.31)	<0.001	1.15 (1.08–1.23)	<0.001
**Rectal cancer**	1.27 (1.09–1.47)	<0.01	1.30 (1.11–1.51)	<0.001
**Lymphoma**	1.70 (1.57–1.85)	<0.001	1.75 (1.61–1.91)	<0.001
**Pancreatic cancer**	1.72 (1.56–2.55)	<0.01	1.80 (1.20–2.70)	<0.01
**Stomach cancer**	1.73 (1.40–2.14)	<0.001	1.92 (1.54–2.38)	<0.001
**Leukemia**	1.81 (1.59–2.05)	<0.001	1.84 (1.62–2.09)	<0.001
**Esophagus cancer**	1.82 (1.39–2.38)	<0.001	1.81 (1.38–2.38)	<0.001
**Lung cancer**	2.43 (2.21–2.68)	<0.001	2.51 (2.28–2.77)	<0.001
**Liver cancer**	2.78 (1.98–3.91)	<0.001	2.95 (2.08–4.17)	<0.001
**Treatments**				
**Primary prostate cancer and RP**	Ref	–	–	–
**Secondary RP**	2.20 (2.02–2.40)	<0.001	2.25 (2.06–2.45)	<0.001
**Primary prostate cancer and EBRT**	Ref	–	–	–
**Secondary EBRT**	1.56 (1.48–1.65)	<0.001	1.59 (1.51–1.68)	<0.001
**Primary prostate cancer and no local treatment**	Ref	–	–	–
**Secondary no local treatment**	1.53 (1.47–1.59)	<0.001	1.53 (1.47–1.59)	<0.001
**Time intervals**				
**Primary prostate cancer**	Ref	–	–	–
**Secondary cancer 7–36 months prior to prostate cancer**	1.92 (1.83–2.02)	<0.001	1.95 (1.85–2.05)	<0.001
**Secondary cancer 37–60 months prior to prostate cancer**	1.77 (1.67–1.88)	<0.001	1.74 (1.64–1.85)	<0.001
**Secondary cancer 61–120 months prior to prostate cancer**	1.58 (1.50–1.67)	<0.001	1.61 (1.53–1.70)	<0.001
**Secondary cancer >120 months prior to prostate cancer**	1.34 (1.27–1.42)	<0.001	1.32 (1.24–1.40)	<0.001

HR, hazard ratio; CI, confidence interval.

### Survival Analyses After 1:4 Propensity Score Matching According to Local Treatment Type: RP *vs*. EBRT *vs*. No Local Treatment

Subsequently, we repeated Kaplan-Meier and Cox regression analyses, after stratification according to local PCa treatment type in patients treated with RP or EBRT or no local treatment (NLT) across all primary cancer types. Here, presence of secondary PCa resulted in worse OM, relative to primary PCa patients. Specifically, 10-year OM rates were respectively 22.1% *vs*. 11.7%, 47.4% *vs*. 36.5%, and 75.3% *vs*. 51.7% after RP, EBRT, or NLT in secondary *vs*. primary PCa patients (**Figures 2B–D**). In multivariable Cox regression models, the respective HRs were 2.3 after RP, 1.6 after EBRT, and 1.5 after NLT in secondary PCa patients, relative to primary PCa patients (**Table 3**, all <0.01).

### Survival Analyses After 1:4 Propensity Score Matching According to Primary Cancer Type

**Figure 3:**
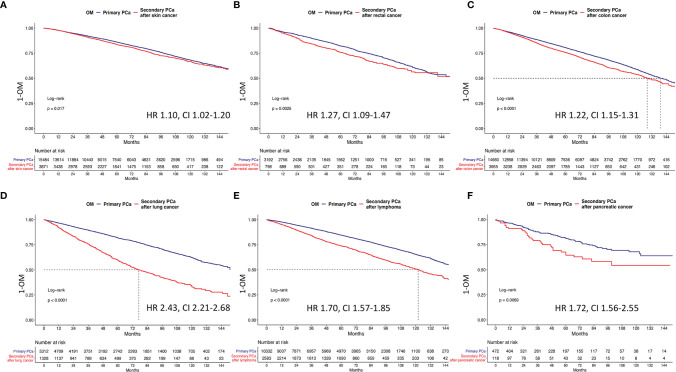
Kaplan-Meier plots depicting overall mortality (OM) for primary and secondary prostate cancer after **(A)** primary skin cancer, **(B)** primary rectum cancer, **(C)** primary colon cancer, **(D)** primary lung cancer, **(E)** primary lymphoma, and **(F)** primary pancreatic cancer. HR, hazard ratio; CI, confidence interval.

**Figure 4:**
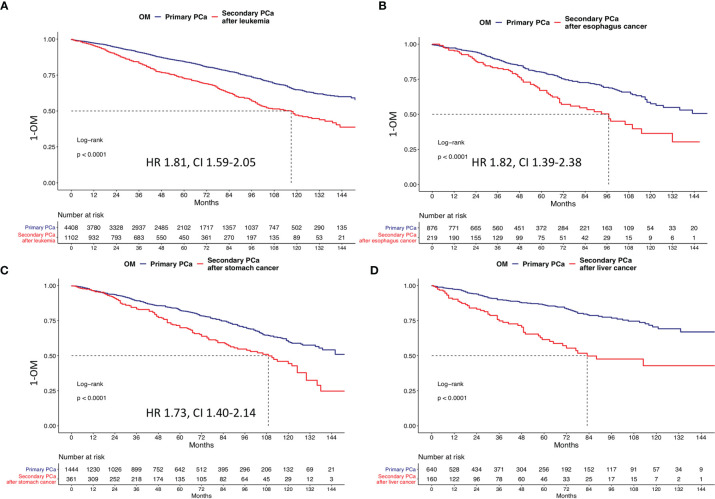
Kaplan-Meier plots depicting overall mortality (OM) for primary and secondary prostate cancer after **(A)** primary leukemia, **(B)** primary liver cancer, **(C)** primary stomach cancer, and **(D)** primary esophagus cancer. HR, hazard ratio; CI, confidence interval.

Kaplan-Meier plots showed in secondary PCa patients with skin, rectal, pancreas, colon, lymphoma, leukemia, stomach, liver, esophagus, and lung cancer *vs*. for primary PCa patients respectively 10-year OM rates of 33.6% *vs*. 32.1%, 43.7% *vs.* 39.3%, 45.7% *vs.* 32.2%, 46.4% *vs.* 41.7%, 49.3% *vs.* 34.8%, 52.9% *vs.* 35.2%, 55.6% *vs.* 40.1%, 57.1% *vs.* 29.5%, 63.7% *vs.* 42.5%, and 67.0% *vs.* 37.9% (**Figures 3** and **4**). All secondary PCa patients harbored a significant OM disadvantage relative to primary PCa patients. The specific multivariable HRs were 1.2, 1.3, 1.8, 1.2, 1,8, 1.8, 1.9, 3.0, 1.8, and 2.5 for respectively secondary PCa patients with primary skin, rectal, pancreas, colon, lymphoma, leukemia, stomach, liver, esophagus, and lung cancer (all *p* < 0.01; **Table 3**).

The proportions of patients that died of secondary PCa (**Table 2**) ranged from 9.8% (in primary lung cancer patients) to 25.7% (in primary rectal cancer patients). Similarly, the proportions of patients that died of primary cancers ranged from 16.4% (skin cancer) to 50.0% (liver cancer). Unfortunately, these cancer-specific rates could not be translated into Kaplan-Meier-derived actuarial estimates due to unavailable time to death.

### Survival Analyses After 1:4 Propensity Score Matching According to Time Interval Length Since Initial Cancer Diagnosis and Secondary PCa Diagnoses

**Figure 5:**
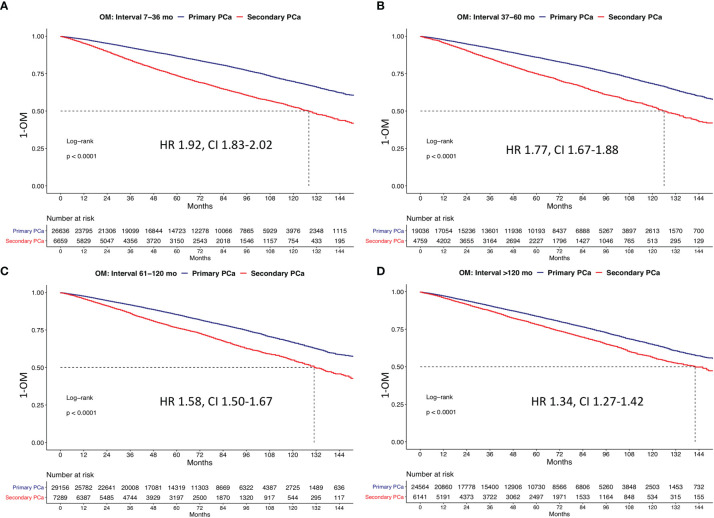
Kaplan Meier plots depicting overall mortality (OM) for primary and secondary prostate cancer according to the time interval between primary cancer and secondary prostate cancer at **(A)** 7–46 months, **(B)** 37–60 months, **(C)** 61–120 months, and **(D)** >120 months. HR, hazard ratio; CI, confidence interval.

Time interval length since initial cancer and secondary PCa diagnoses was stratified into four groups between 7 and 36 (*n* = 6,659) *vs.* 37 and 60 (*n* = 4,759) *vs.* 61 and 120 (*n* = 7,289) *vs.* ≥121 months (*n* = 6,141). In Kaplan-Meier plots (**Figure 5**) that addressed the comparison between secondary PCa diagnosed between 7 and 36 months after primary cancer diagnosis, relative to primary PCa, the respective 10-year OM rates were 47.4% *vs.* 30.4%. These OM rates translated into a multivariable HR of 1.95. The subsequent stratifications (37–60 *vs.* 61–120 *vs.* ≥121 months) resulted in 10-year OM rates in secondary PCa patients of 47.4% *vs.* 31.8%, 45.1% *vs.* 32.3%, and 44.0%% *vs.* 35.2% months in primary PCa patients. The respective multivariable HR for 7–36 *vs.* 37–60 *vs.* 61–120 *vs.* ≥121 months were 1.7, 1.6, and 1.3.

## Discussion

We hypothesized that secondary PCa patients will harbor less favorable disease characteristics in addition to exhibiting less favorable prognosis, relative to primary PCa patients. To test this hypothesis, we identified 24,848 secondary PCa patients and 531,732 primary PCa patients, for the purpose of comparisons. Here, secondary PCa patients were older than their primary PCa counterparts. On average, secondary PCa diagnosis (69 years) was made 6 years after primary cancer diagnosis (63 years). Moreover, age at diagnosis variability was also recorded according to primary cancer type in secondary PCa patients. The latter ranged from 66 (liver cancer) to 72 years (colon cancer). These observations are different from the more historical reports about secondary PCa. For example, in the study by Dinh et al., median age in patients with secondary PCa diagnosis was 73, which is significantly older than in the current study (17). It may be postulated that a selection bias is operational regarding the age at secondary PCa diagnosis. The latter may be directly related to aggressiveness and mortality probability of the primary cancer diagnosis. Although such simplified explanation is attractive, several confounding variables may be operational. For example, patients with most aggressive cancers may be expected to be never be diagnosed with secondary PCa. Conversely, long-term survivors of highly aggressive primary cancer variants may still be diagnosed with secondary PCa. The latter may render generalizations about the effect of aggressive primary cancer on rates and ages at secondary PCa diagnosis virtually uninterpretable.

Less pronounced differences were recorded in PSA distributions of secondary and primary PCa patients, evidenced by respectively 6.9 (IQR 4.9–11.5) *vs.* 6.6 ng/ml (IQR 4.8–10.6) PSA values at diagnoses. Additionally, small differences in PSA at diagnoses were recorded in secondary PCa patients, according to primary cancer type and ranged from 6.5 (skin cancer) to 7.8 ng/ml (pancreatic and liver cancers). Similarly, we also observed small differences in Gleason grade groups IV and V. Here, secondary PCa patients exhibited less favorable grade. This observation is in an agreement with previous publications, where secondary PCa patients also harbored higher rates of Gleason grade group IV/V (18, 19). Finally, no clinically meaningful differences were identified according to stage. Taken together, these data indicate that despite more advanced age and small disadvantage in PSA at diagnosis and PCa grade, secondary PCa patients do not exhibit crucial PCa characteristic differences at initial diagnosis. However, this interpretation may be biased and warrants methodologically more stringent analyses. This suspicion prompted the use of propensity score matching, according to age as well as patient and PCa characteristics. Moreover, we also applied additional multivariable adjustment in all subsequent survival analyses. The intent was to most thoroughly test for prognostic differences with strictest reduction of bias and/or confounding.

In part 1 of the OM analyses, the propensity-matched comparisons addressed the entire cohort of secondary PCa patients, relative to all primary PCa controls. In part 2 of OM analyses, we examined the effect of primary and secondary PCa in respectively RP-, EBRT-, and NLT-treated patients. In the third part of the analyses, we sequentially compared secondary PCa patients, relative to their primary PCa counterparts, according to the type of primary malignancy diagnosed prior to secondary PCa. In the fourth part of analyses, we stratified the comparisons according to the length of the time interval between primary cancer and secondary PCa diagnoses.

In 1:4 matched survival analyses that addressed the entire secondary PCa population, relative to their primary PCa controls, we identified pronounced survival disadvantage in secondary PCa patients (10-year OM 46% *vs.* 35.7%). A similar absolute and relative magnitude of the survival disadvantage in secondary PCa patients was also recorded in subgroup analyses of RP-, EBRT-, and NLT-treated patients. In the third part of the analyses, we invariably recorded a survival disadvantage in all secondary PCa patients diagnosed with the 10 most common nonurological initial cancers (HRs: 1.1–2.8). These observations are consistent with previous findings. For example, Klippstein et al. also investigated a survival disadvantage (overall and cancer-specific survival) of 1,552 secondary PCa patients, relative to primary PCa patients (19). However, due to sample size limitations, no primary cancer-specific analyses could be conducted in these analyses and should be ideally performed in further multi-institutional analyses.

Taken together, the above findings indicate that despite apparently small to no differences in patient and/or PCa characteristics at baseline between secondary and primary PCa patients, very important survival disadvantages were applied to secondary PCa patients. This observation was made despite most stringent and methodologically strict statistical matching and multivariable adjustment. In consequence, the persistence of this disadvantage across therapy types suggest that secondary PCa patient harbor a prognostic disadvantage, relative to primary PCa patients, despite exhibiting almost the same baseline characteristics. The observed disadvantage applies across all primary cancer types and persists regardless of primary treatment type (RP and EBRT) and also after further multivariable adjustment for Gleason grade group and PSA. In consequence, the detrimental effect of secondary PCa appears robust and generalizable. The observation of Zhu et al. validates our hypothesis about the aggressiveness of primary cancer that may impact, as well as determine the natural history of treated secondary malignancies (24). The above findings, especially that with longer time interval between primary cancer and secondary PCa life expectancy approximates the life expectancy to primary PCa, should be considered treatment decision making, when secondary PCa patients are counseled.

Finally, in analyses according to length of time interval between primary cancer and secondary PCa diagnoses, we observed that the survival disadvantage decreases with increasing length of time. This observation may indicate that in individuals in whom the time between initial and secondary cancer diagnoses is lengthy, the secondary PCa phenotype may be more comparable with primary PCa. Conversely, when the length of interval between primary cancer and secondary PCa is short, the phenotype might be more aggressive, as evidenced by greater survival disadvantage. We are the first to report this observation, which should be validated in other large-scale databases.

Our observations imply that patients with secondary PCa should be given more careful consideration to eliminate the survival disadvantages that we recorded. Unfortunately, the nature of our data does not allow to identify whether the increase in OM in secondary PCa patients, relative to their primary PCa counterparts, was related to the primary cancer or secondary PCa. In consequence, measures aimed at reducing this survival disadvantage of secondary PCa patients should not only focus on PCa treatments and follow-up but also on treatments and follow-up of their primary cancer. Finally, more detailed databases would allow to distinguish between mortality from primary or secondary cancer could help fine tuning further research and clinical management.

Our work has limitations and should be interpreted in the context of its retrospective and population-based design. Second, the nature of our data does not allow to define specific mortality time points to estimate Kaplan-Meier actuarial mortality rates. This limitation is shared with all previous publications focusing on secondary cancers, after specific primary cancers in large-scale databases (24–26). Limited stage and grade information was available for each of the 10 examined primary cancers and matching could not be performed for PSA and Gleason grade group without losing secondary PCa patients. Finally, important variables such as performance status and comorbidities are not available in the SEER database (27). These also contribute to OM rates but could neither be addressed in the current study or in previous analyses (24–26).

## Data Availability Statement

The raw data supporting the conclusions of this article will be made available by the authors, without undue reservation.

## Ethics Statement

Ethical review and approval was not required for the study on human participants in accordance with the local legislation and institutional requirements. Written informed consent for participation was not required for this study in accordance with the national legislation and the institutional requirements.

## Author Contributions

Conceptualization: MW, LN, CR, FC, and PK. Methodology: MW and ZT. Formal analysis and investigation: MW, CW, and ZT. Writing (original draft preparation): MW, LN, FC, and PK. Writing (review and editing): FS, ABr, DT, MG, ABe, FR, and FC. Supervision: FS, ABr, FR, FC, and PK. All authors contributed to the article and approved the submitted version.

## Conflict of Interest

The authors declare that the research was conducted in the absence of any commercial or financial relationships that could be construed as a potential conflict of interest.

## Publisher’s Note

All claims expressed in this article are solely those of the authors and do not necessarily represent those of their affiliated organizations, or those of the publisher, the editors and the reviewers. Any product that may be evaluated in this article, or claim that may be made by its manufacturer, is not guaranteed or endorsed by the publisher.
